# Proteomics of the corpus callosum to identify novel factors involved in hypomyelinated Niemann-Pick Type C disease mice

**DOI:** 10.1186/s13041-019-0440-9

**Published:** 2019-03-11

**Authors:** Fan Yang, Yudong Guan, Xiao Feng, Arndt Rolfs, Hartmut Schlüter, Jiankai Luo

**Affiliations:** 1 Albrecht-Kossel-Institute for Neuroregeneration, University Medical Center Rostock, Gehlsheimer Strasse 20, 18147 Rostock, Germany; 2 Institute of Clinical Chemistry & Laboratory Medicine, University Medical Center Hamburg-Eppendorf, 20246 Hamburg, Germany

**Keywords:** Proteomics, Corpus callosum, Npc1, Myelination, Lipid transport, Gltp

## Abstract

Hypomyelination in the central nerves system (CNS) is one of the most obviously pathological features in Niemann-Pick Type C disease (NPC), which is a rare neurodegenerative disorder caused by mutations in the NPC intracellular cholesterol transporter 1 or 2 (Npc1 or Npc2). Npc1 plays key roles in both neurons and oligodendrocytes during myelination, however, the linkage between the disturbed cholesterol transport and inhibited myelination is unrevealed. In this study, mass spectrometry (MS)-based differential quantitative proteomics was applied to compare protein composition in the corpus callosum between wild type (WT) and NPC mice. In total, 3009 proteins from both samples were identified, including myelin structural proteins, neuronal proteins, and astrocyte-specific proteins. In line to hypomyelination, our data revealed downregulation of myelin structural and indispensable proteins in Npc1 mutant mice. Notably, the reduced ceramide synthase 2 (Cers2), UDP glycosyltransferase 8 (Ugt8), and glycolipid transfer protein (Gltp) indicate the altered sphingolipid metabolism in the disease and the involvement of Gltp in myelination. The identification of most reported myelin structural proteins and proteins from other cell types advocates the use of the corpus callosum to investigate proteins in different cell types that regulate myelination.

**Electronic supplementary material:**

The online version of this article (10.1186/s13041-019-0440-9) contains supplementary material, which is available to authorized users.

## Introduction

Mutations of either NPC intracellular cholesterol transporter 1 or 2 (Npc1 or Npc2) cause the Niemann-Pick Type C disease (NPC), which is a rare recessive neurological disorder [1, 2]. The disease exhibits a massive accumulation of cholesterol and other lipids in the late endosome and lysosome (LE/LY) [3]. Neuron loss and hypomyelination in the central nervous system (CNS) are the most obvious pathological features in patients and the mouse model of the disease [4–7]. The NPC mouse (BALB/cNctr-Npc1m1N/J), carrying a spontaneous mutation of *npc1* without functional Npc1 protein, is frequently used as the mouse model for NPC disease. Myelin disturbance has been reported in NPC mice in the 1980s [8]. Takikita et al. have described hypomyelination in the brain of NPC mice and proposed that disturbed myelination contributes to the axonal injury [6]. The arrested oligodendrocyte maturation and delayed myelination in conditional *npc1*-knockout neurons or oligodendrocytes conclude the essential role of *npc1* in both neurons and oligodendrocytes during myelination [9]. Our previous study also confirms a delayed and reduced myelination in the corpus callosum of NPC mice with an unaltered number of oligodendrocytes, but their maturation is inhibited [10].

A high level of cholesterol is essential for myelination, as indicated by delayed myelination in oligodendrocytes with a conditional mutation of squalene synthase (SQS) [11]. Impaired cholesterol transport from the LE/LY presumably causes a cholesterol shortage in other cellular compartments, such as distal axons in NPC disease [12], however, lovastatin–a cholesterol synthesis inhibitor restores myelination in the cultivated NPC oligodendrocytes, proving that cholesterol accumulation in the LE/LY rather than the shortage in distal axons causes hypomyelination in NPC disease [10]. Similarly, lipid accumulation induces myelin disturbance has been reported in many lysosomal storage diseases [13].

Besides lipids, myelin sheaths contain variously specific proteins. By proteomic analyses of the myelin-enriched fraction from mice, 92 proteins have been identified by Roth et al. and 344 proteins by Jahn et al. [14, 15]. In the proteomes of the mouse and human, 259 commonly identified proteins from myelin fractions have been confirmed [16]. Furthermore, a few myelination-related transcription factors are identified, such as oligodendrocyte transcription factor 1 (Olig1), Olig2, homeobox protein Nkx-2.2 (Nkx2.2), SRY-related HMG-box 10 (Sox10), and myelin gene regulatory factor (Myrf), which regulate the expression of major myelin proteins [17–20]. Our previous study reported the reduced expression of myelin basic protein (Mbp), proteolipid protein (Plp) and myelin-oligodendrocyte glycoprotein (Mog), and downregulation of Olig1 and Olig2 in the corpus callosum, suggesting a hypomyelination in NPC mice [10].

To further investigate hypomyelination in NPC disease, in this study the mass spectrometry (MS)-based differential quantitative proteomics was used to compare the protein composition in corpora callosa between WT and NPC mice. The results showed that not only most of the reported myelin proteins but also 21 significantly differential expression proteins between NPC and WT have been identified. Most of the downregulated proteins are myelin proteins, including breast carcinoma-amplified sequence 1 (Bcas1), ectonucleotide pyrophosphatase (Enpp6), Mbp, and UDP glycosyltransferase 8 (Ugt8), which are the indispensable myelin proteins. Notably, our data revealed downregulation of 3 sphingolipid-related proteins: Cers2, Ugt8, and Gltp, indicating an altered sphingolipid metabolism in the disease and the involvement of Gltp during myelination. Besides the reported myelin proteins, we identified proteins from other cell types that participant in myelination, e.g. from neurons, astrocytes, and microglia. Therefore, our data suggest that the corpus callosum can be used to investigate molecular dynamics and signal cascades among different cell types during myelination.

## Materials and methods

### Separation of the corpus callosum

**Fig. 1:**
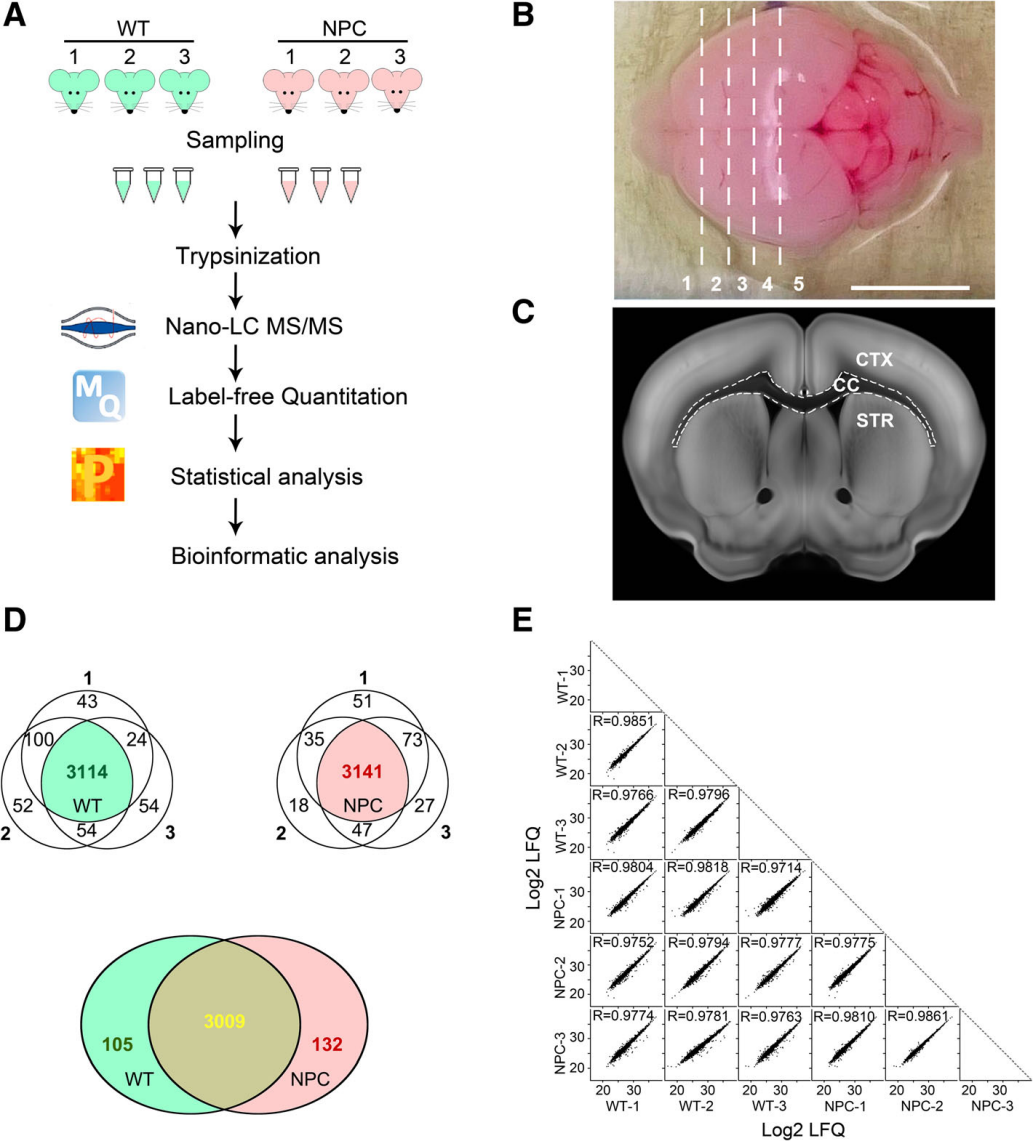
Comparison of protein patterns in corpora callosa of wildtype and Npc1 mutant mice at P12. **a**: The schematic diagram of the workflow for proteomic analysis from 3 biological replicants of wildtype (WT) and Npc1 mice (NPC). **b**, **c**: Separated brain was cut according to the dashed lines in the forebrain and the corpus callosum (CC) in the transaction was separated with the cortex (CTX) and the striatum (STR) from sections 2 to 4 in **b**. Scale bar: 1 cm in **b**. **c** was adapted from the Allen Brain Atlas (http://atlas.brain-map.org/). **d**: The Venn diagram of identified proteins from each sample. Valid proteins from each genotype were illustrated in green for WT samples and in red for NPC samples. The common proteins from both WT and NPC were in yellow. **e**: The Scatter plots of Log2 LFQ values of identified proteins between samples and the Pearson correlations were calculated (the values of R)

Heterozygous Npc1 mice (BALB/cNctr-Npc1m1N/J) were purchased from the Jackson Laboratories and used to produce NPC and WT mice. All experiments were approved by the local ethical committee and conducted according to the guidelines for the Care and Use of Laboratory Animals. Mice were genotyped and sacrificed at postnatal day (P) 12 for tissue preparation. At least three animals from each genotype were used for proteomic analysis (Fig. 1a) and another 3 pairs for Western blots. The isolated forebrains were transected and corpora callosa were dissected from adjacent tissues (Fig. 1b and c). Tissues were frozen immediately in liquid nitrogen and stored at − 80 °C.

### Sample preparation for mass spectrometric analysis

Tissues were frozen by liquid nitrogen and ground rigorously by pre-chilled flame-polished glass rods. The ground samples were homogenized in 150 μl of 8 M urea and 50 μl of SDC buffer (1% SDC in 0 .1M TEAB) and sonicated on ice. Protein concentration was measured by the BCA Protein Assay (Thermo Fisher, #23225). Samples (100 μg protein from each sample) were digested by filter-aided sample preparation (FASP) [21]. Basically, samples were placed in 10 k centrifugal filter units (Amicon Ultra-0.5 Centrifugal Filter Unit) and the buffer was exchanged by 6 M urea, reduced by 20 mM dithiothreitol (DTT) and alkylated by 40 mM iodoacetamide (IAA), then digested by trypsin (Promega, the enzyme to protein ratio is 1:50) in 400 μl of 100 mM ammonium bicarbonate buffer at 37 °C for 20 h. The digested peptides were collected in water, lyophilized and stored at − 20 °C for mass spectrometric analysis.

### NanoLC-MS/MS analysis and data analysis

The tryptic peptides were dissolved in 0.1% formic acid (FA) and loaded into the nanoAcquity Ultra Performance liquid chromatography (UPLC) system (Waters, USA). A C18 trapping column (Waters, 180 μm × 20 mm, 5 μm, 100 Å) and an analytical C18 column (Thermo Fisher Scientific™ Acclaim PepMap™ RSLC, 75 μm × 25 cm, 2 μm, 100 Å, USA) were used to separate peptides. The UPLC was coupled with Q Exactive Hybrid Quadrupole-Orbitrap Mass Spectrometer (Thermo Fisher Scientific™). The positive voltage was set to 1.8 kV; the scan range m/z was 375–1600 Th; the collision energy of HCD was 27%; the MS2 acquisition was in the data-dependent mode by top 10. The data were processed by the Thermo Xcalibur 4.0.27.13 (Thermo Fisher Scientific™). The database searching and label-free quantification (LFQ) were performed by MaxQuant platform (Version No. 1.6.2.3, http://www.coxdocs.org/doku.php?id=maxquant:start). All the raw files were searched with mouse proteome sequences from UniProt. The “unique plus razor peptides” was chosen for protein quantification [22]. The precursor mass tolerance was set to 20 ppm; the fragment mass tolerance was 0.5 Da; dynamic modification included oxidation (15.995 Da), acetyl (42.011 Da) and fixed modification carbamidomethyl (57.021 Da).

### Data analysis and comparison with other proteomic data

Data was uploaded into Perseus software and the online manuals were followed (Version No. 1.6.2.1, http://www.coxdocs.org/doku.php?id=perseus:start). Basically, the proteins labeled by only identified by site, reverse, and potential contaminant were removed from the data and the proteins were valid if they were identified from all samples in WT or NPC group. Pearson’s correlation was calculated with the default setting from the software. To obtain the significantly expressed proteins, two-sample student’s t-test was used, and S0 of 1 and false discovery rate (FDR) of 0.05 were set.

To elucidate the function of the identified proteins, the valid proteins were analyzed by the gene ontology cellular compartment term enrichment (Go CC; GOTERM_CC_DIRECT) from DAVID online tool (https://david.ncifcrf.gov/). The differentially expressed proteins were uploaded to the Gene Ontology Consortium (http://geneontology.org/) for the Go CC, biological processes (Go BP), and Reactome pathway (Reactome) analysis, both the protein count and FDR value of each term were obtained. The background of all analyses is based on all proteins of *Mus musculus* and the terms with FDR below 0.05 were listed.

Additionally, our data were compared with the published data from John et al. [15] and from the supplement Table-2 (https://www.nature.com/articles/nn.4160#supplementary-information) reported by Sharma et al. [23] to verify our results and assign the cell type specificity of identified proteins, respectively. All the histogram, Pie chart, and the Venn diagram were produced by excel.

### Western blot analysis

The ground samples (produced by the same procedure as in MS) were homogenized in the RIPA buffer with the complete protease inhibitor cocktail (Roche 4,693,159,001). SDS-PAGE was performed in the 8–16% Criterion TGX Precast Midi Protein Gels (Bio-rad, #567–1084) and proteins were blotted to Midi Nitrocellulose membranes (Bio-rad, #17001915) by the Trans-Blot Turbo Transfer System (Bio-rad, #17001915). The antibodies against Mbp (Biolegend, SMI-99, 1: 1000), Gltp (Santa Cruz, SC-514289, 1:200), Enpp6 (Thermo Fisher, PA5–25140, 1:500), Bcas1 (Sigma-Aldrich, SAB2900809, 1:500), and GAPDH (Abcam, ab8245, 1:10,000) were applied to the membrane. IRDye-680/800 conjugated secondary antibodies (Rockland, #926–68,021, #610–131-121, 1:10,000) were used to visualize the detected proteins by the Odyssey Infrared Imaging System. Semi-quantitative analysis was performed by Image Studio Lite 4.0. Data were reported as mean ± SEM from three independent experiments. Student’s t-test was calculated in MS Excel software. A difference was considered as significant when the *p*-value was less than 0.05 (*p* < 0.05).

## Results

### Similar proteomic patterns in the corpora callosa between WT and NPC mice

To unravel factors that are involved in hypomyelination in NPC disease, three replicates of corpora callosa from either WT or NPC mice at P12 were separated for NanoLC-MS/MS analysis (Fig. 1a-c). We identified 3281, 3320 and 3246 proteins from the three WT samples, and 3300, 3241 and 3288 proteins from NPC samples, respectively. In the WT samples, 3114 proteins were identified in all replicants and in the NPC 3141 proteins (green and pink color in the Venn diagram; Fig. 1d). Furthermore, 3009 proteins were confirmed in both groups (yellow in Fig. 1d), while 105 proteins only in WT and 132 only in NPC. Noted that most of the proteins identified only in one group were the low abundant proteins (the lowest 30%) and the exclusion of these proteins was due to the limited resolution by the current proteomic approaches. Therefore, the proteins detected in both WT and NPC mice were used for analysis (Additional file 1: Table S1).

To estimate the quality of our data, the Pearson correlation was calculated. The coefficients between replicants were above 0.97 in both WT and NPC groups, demonstrating the technical reliability of our analyses (Fig. 1e). Unexpectedly, the coefficients were only slightly different between WT and NPC samples when compared to them each other (Fig. 1e), suggesting similar proteomic patterns between them, although myelination was significantly disrupted in the corpus callosum in NPC mice [10].

### The identification of reported myelin proteins

**Fig. 2:**
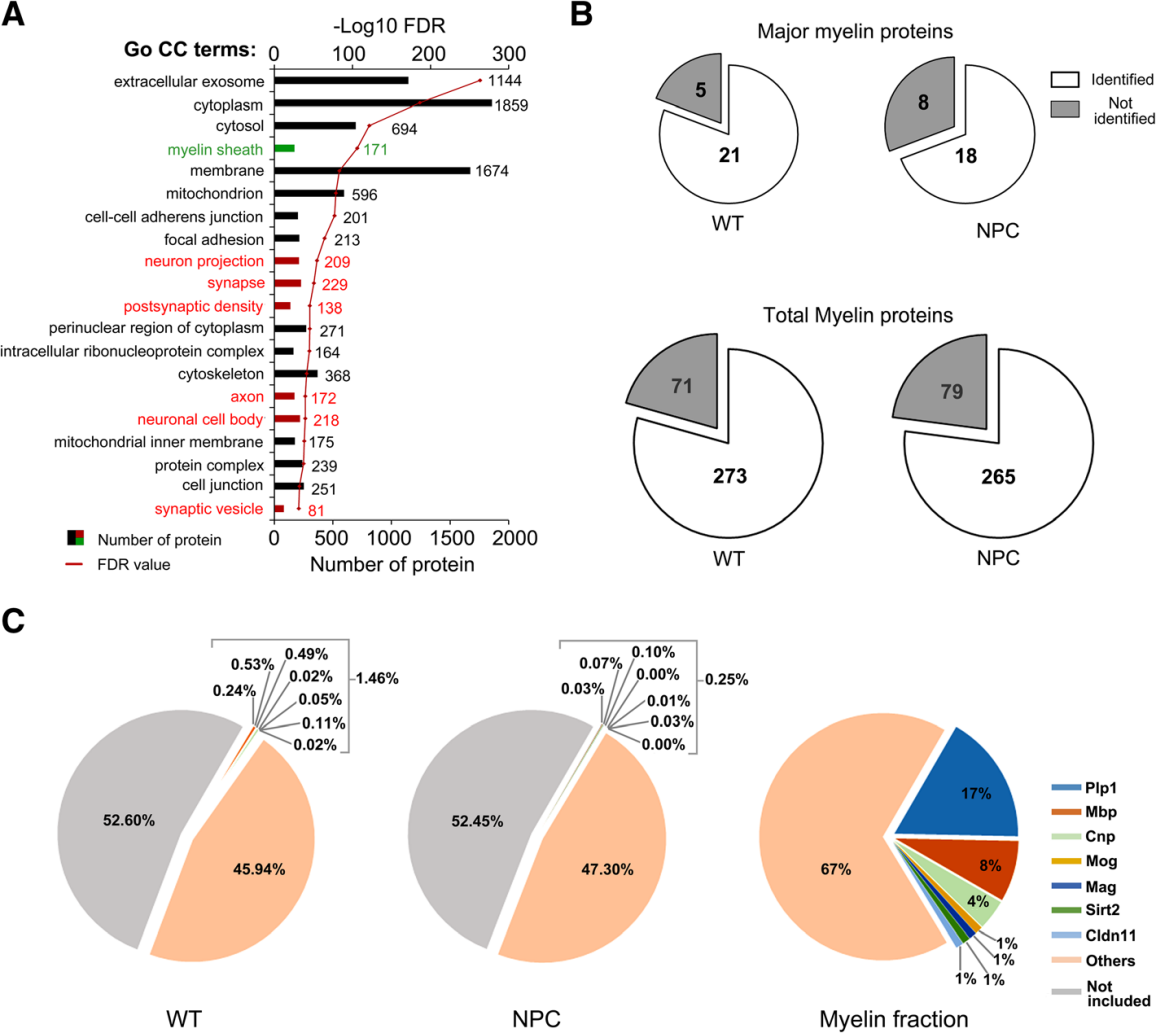
Function analyses of identified proteins. **a**: The gene ontology cellular compartment term (Go CC) enrichment of identified proteins. The myelin-specific term was labeled in green and neuron-specific terms in red. The number in the bars indicated protein number in each term, the red point marked FDR value presented by its -Log10 value. **b**: The numbers of identified proteins were compared to the protein data from myelin-enriched fraction reported by Jahn et al. (2009). The numbers of identified major or total myelin proteins were illustrated in white and non-identified in grey. **c**: The abundance of identified proteins from WT, NPC, and the data derived from myelin-enriched fraction as reported by Jahn et al. (2009). The sums of valid protein abundances from each group were set to 100%. The top 7 proteins from myelin-enriched fraction were labeled individually with different colors. Proteins that not reported from myelin-enriched fraction were labeled in grey

To categorize the identified proteins, Go CC analysis was performed and the top 20 terms with the lowest FDR values were listed (Fig. 2a). Besides the regularly cellular compartments, such as the extracellular exosome, cytoplasm, membrane, and mitochondrion, various proteins were categorized into cell-type specific compartments: a total of 171 proteins were classified into the term of myelin sheaths (green in Fig. 2a); 209 proteins into the neuron projection, 229 proteins into the synapse, and 172 proteins into the axon (red in Fig. 2a). In addition, astrocyte-specific proteins [23] including glial fibrillary acidic protein (Gfap), aquaporin-4 (Aqp4), and cytosolic 10-formyltetrahydrofolate dehydrogenase (Aldh1l1) were also identified. The results demonstrated the anatomical features of the corpus callosum, where not only myelin sheaths and axons, but also cell bodies of oligodendrocytes, astrocytes, and microglia were located.

Jahn et al. have summarized a total of 344 proteins, including 26 well-known myelin proteins and 318 myelin-associated proteins, from the myelin-enriched fraction of the mouse CNS [15]. Similarly, most of these proteins were identified in this study, including 21 myelin proteins and 273 myelin-associated proteins in the WT group and 18 myelin proteins and 265 myelin-associated proteins in the NPC (Fig. 2b). Furthermore, the relative abundance of the identified myelin proteins was evaluated by comparing their LFQ intensities with the sum of all valid proteins in each group. Because the proteins of other cellular compartments and from other cell types were included in our data, the amount of identified myelin proteins consisted of only 47.40% (45.94% + 1.46%) in the WT group and 47.55% (47.3% + 0.25%) in the NPC of the total identified proteins from the corpus callosum (Fig. 2c). The amount of the most abundant myelin proteins, such as Plp1 (17%), Mbp (8%), 2′,3′-cyclic-nucleotide 3′-phosphodiesterase (Cnp; 4%), Mog (1%), myelin-associated glycoprotein (Mag; 1%), NAD-dependent protein deacetylase sirtuin-2 (Sirt2; 1%), and Claudin-11 (Cldn11; 1%), is about 33% of the total proteins in the myelin-enriched fraction as estimated by Jahn et al. (Fig. 2c) [15]. The sum of these high abundant myelin proteins composed only 1.46% for all the identified proteins of the corpus callosum in WT mice, while only 0.25% in the NPC mice (Fig. 2c). The reduced amount of myelin proteins in the NPC group when compared to WT, was consistent with hypomyelination in the NPC mice.

### Differentially expressed proteins in NPC mice

**Fig. 3:**
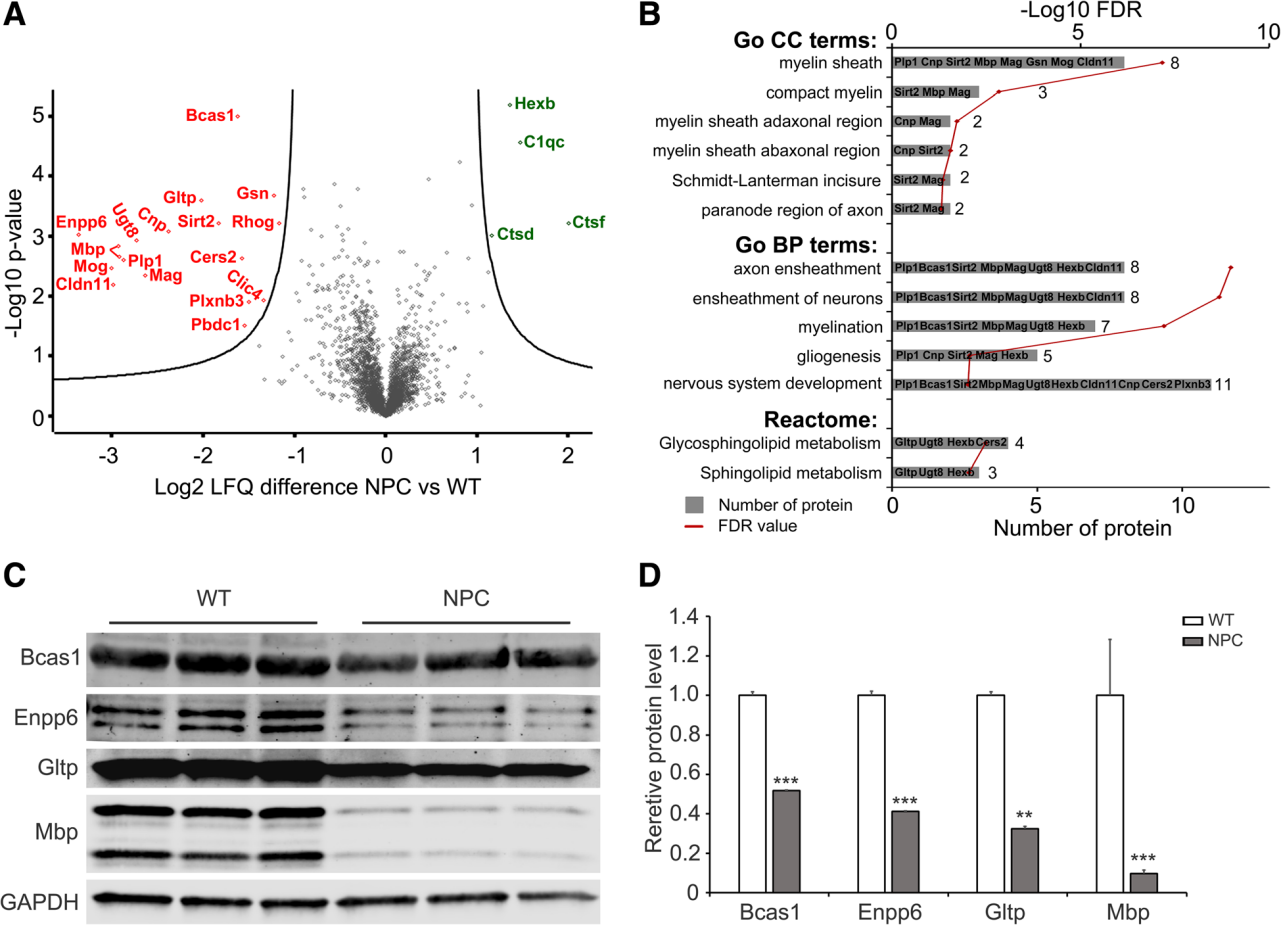
Differentially expressed proteins between WT and NPC mice. **a**: Volcano plot of the Log2 LFQ differences versus -Log10 *P*-values of the common proteins between WT and NPC. The downregulated proteins in NPC were on the left side and significant ones were labeled in red; the upregulated proteins were on the right and significant ones were labeled in red. **b**: The Go CC, biological processes (Go BP) and Reactome pathway (Reactome) enrichment of differentially expressed proteins, the names of protein in each term were listed on the bars, the red point marked FDR value presented by its -Log10 values. **c**, **d**: Western blots (**c**) and quantification (**d**) of the differentially expressed proteins from corpus callosum of mice. The value of each protein was normalized to the corresponding loading control and the values of WT were set to 1. Three biological replicants were compared and GAPDH was used as the loading control. Student’s t-test was used. *** *p* < 0.001

**Fig. 4:**
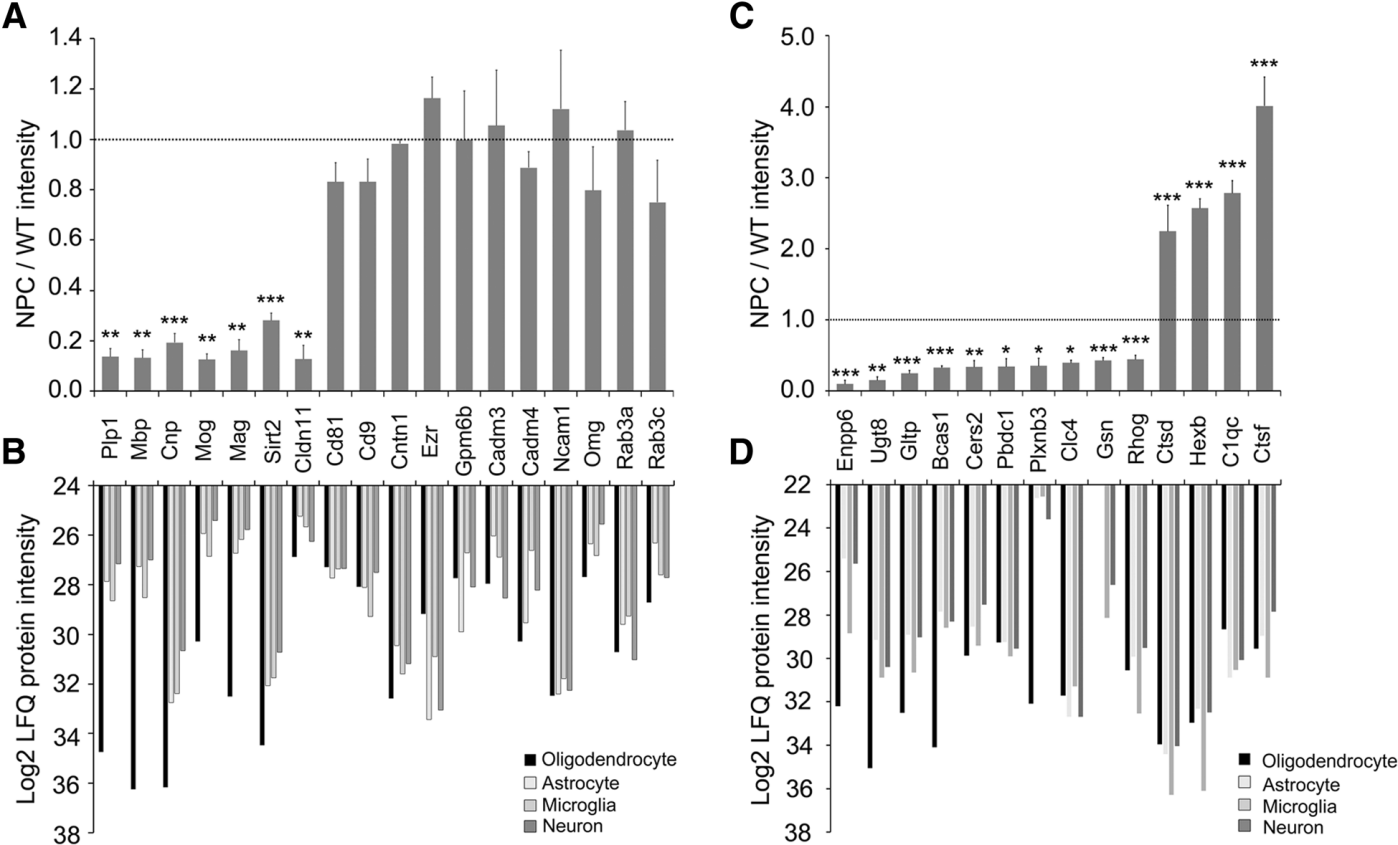
The cell type specificity of the myelin proteins and differentially expressed proteins. **a**, **c**: The comparison of the LFQ intensities of the identified myelin proteins between WT and NPC mice. The LFQ intensities of NPC were normalized to WT and the WT LFQ intensity is set as 1, indicated by the dotted line. Student’s t-test was used. * *p* < 0.05, ** *p* < 0.01, *** *p* < 0.001. **b**, **d**: The cell-type specific expression patterns of myelin proteins and the differently expressed proteins were analyzed based on the proteomic data reported by Sharma et al. (2015)

Although the protein profiles exhibited high similarities between WT and NPC mice, the expressions of 21 proteins were significantly different (the 2 isoforms of Mbp were combined in this study), in which 17 proteins were downregulated (red in Fig. 3a) and 4 proteins upregulated in NPC mice (green in Fig. 3a). The Go CC analysis indicated that the enrichment of myelin structure proteins, such as Plp1, Cnp, Sirt2, Mbp, Mag, Mog, gelsolin (Gsn), and Cldn11, were included in the term of the myelin sheath, also some of them were categorized into specific myelin structures, e.g., Sirt2, Mbp, and Mag in the compact myelin, and Sirt2 and Mag in the Schmidt-Lanterman incisure (Fig. 3b). The Go BP analysis exhibited the enrichment of proteins in development, myelination, and gliogenesis. The proteins of Plp1, Bcas1, Sirt2, Mbp, Mag, Ugt8, Hexb, Cldn11, Cnp, Cers2, and plexin-B3 (Plxnb3) were included in the term of nervous system development, while Plp1, Bcas1, Sirt2, Mbp, Mag, Ugt8, Hexb, and Cldn11 also in the axon ensheathment; and Cnp, Sirt2, Mag, Hexb in the gliogenesis (Fig. 3b). Additionally, the proteins of Plp1, Mbp, Cnp, Mog, Mag, Sirt2, Cldn11, Enpp6, Bcas1, Rho-related GTP-binding protein RhoG (Rhog), and Gsn have been reported in the myelin-enriched fraction [15]. Taken together, the twelve-downregulated proteins (Plp1, Mbp, Cnp, Mog, Mag, Sirt2, Cldn11, Enpp6, Bcas1, Rhog, Ugt8 and Gsn) and the upregulated protein (Hexb) in the corpus callosum of NPC mice were in the myelin structures or involved in the regulation of myelination, demonstrating hypomyelination in the disease.

Besides the myelin-related proteins, the Reactome pathway analysis indicated the enrichment of proteins in the sphingolipid metabolism: the proteins of Gltp, Ugt8, Hexb, Cers2 were in the sphingolipid metabolism; Gltp, Ugt8, and Hexb in the glycosphingolipid metabolism. The Protein of PBDC1 (Pbdc1), Plxnb3, and chloride intracellular channel protein 4 (Clc4) were also upregulated, but their functions were not related to myelination. Besides Hexb protein, Ctsf and CtsD are lysosome proteases that participate in intracellular degradation; and C1qc (a subunit of complement C1q) was the significantly upregulated protein in the corpus callosum of NPC mice.

To confirm the results of the proteomic data, we separated the corpus callosum from both WT and NPC mice at P12 and performed Western blot analyses. Our results revealed that the amounts of Bcas1, Enpp6, and Gltp were obviously reduced in the corpus callosum of NPC mice, with only 51.7% of Bcas1, 41.2% of Enpp6, and 32.2% of Gltp in NPC samples when compared to WT at P12 (Fig. 3c, d). The Mbp protein was used as a positive control, which was only 9.8% in NPC mice compared to WT mice (Fig. 3c, d).

## Discussion

### Analyzing myelination in the corpus callosum

To investigate the disturbed signal pathways of myelination in NPC mice, the proteomic analysis of the corpus callosa from P12 mice was performed in the present study. The myelin-enriched fraction from the density gradient centrifugation has been utilized in studies to explicate the protein composition of the myelin, but it contains exclusively compacted myelin sheaths [15, 16] and rarely oligodendrocyte cell bodies and ensheathed axons. However, myelination is coordinated by interactions between neurons and oligodendrocytes and supported by astrocytes [24]. Since the corpus callosum contains multiple cell types, protein changes in other cell types besides oligodendrocytes can also be monitored in the corpus callosum. The absence of 20% proteins identified from myelin-enriched fraction in this study reflexes the limitation of the current proteomics in analyzing complex samples (Fig. 2b). Even though, the results of Go CC analysis and the identification of most proteins reported by John et al. [15] prove the reliability of our data (Fig. 2). Therefore, our results advocate that the corpus callosum or similar structures can aid as an appropriate system for elucidation of protein dynamics and signal cascades in different cell types during myelination.

The corpus callosa from P12 mice were chosen in the present study to avoid the existence of the high-abundant proteins, which challenge proteomic analysis. Because myelination in the corpus callosum starts at around P9 and completes at P40 [10, 25], the relative abundance of top 7 myelin proteins, which constitute about 33% of total protein in myelin-enriched fraction, is only 1.46 and 0.25% of all identified proteins in the corpus callosum from P12 WT and NPC mice, respectively (Fig. 2c). The lower abundance of these proteins is due to the complex cellular composition of the corpus callosum compared to myelin-enriched fraction and also the less enrichment of myelin proteins from P12 versus adult mice. Moreover, the proteins that participate in regulating myelination are easier to be captured in the developmental stages from younger mice than older ones.

### Downregulated myelin structural and indispensable proteins

Among the downregulated proteins, twelve were annotated as myelin proteins, in which some are essential for the axonal integrity and myelination. The deficiency of Bcas1, Enpp6, Mbp, or Ugt8 disrupts myelination and causes hypomyelination in mice [26–28]. While the knockout of Plp1, Cnp, or Mag doesn’t inhibit myelin formation but causes widespread axonal swelling and degeneration [29–32]. The reduced transport of Sirt2 has been reported in the Plp1-knockout mouse [33] and Sirt2-knockout exhibits axonal degeneration and locomotor disability in 13-month old mice [34]. Despite the enrichment of Mog and Cldn11 in the myelin-enriched fraction, the knockout of these proteins doesn’t exhibit clinical or histological abnormalities [35, 36]. The downregulation of essential proteins plausibly leads to disrupted myelination or being the result of hypomyelination in NPC disease, however, its connection to the disturbed cholesterol transport is not axiomatic.

Although Olig1 and Olig2 proteins are not identified in the current results, their downregulation has been found in the corpus callosum of NPC mice by our previous study [10]. Olig1 and Olig2 are oligodendrocyte-specific transcription factors. Ablation of either protein arrests oligodendrocyte in a progenitor stage and reduces the expressions of Mbp, Plp1, and Cnp, causing severe myelin deficits [37, 38]. Olig2 guides Smarca4/Brg1 to bind oligodendrocyte-specific enhancers and increases the expression of myelin proteins [39]. A study identifies several Olig2 binding regions of proteins (Nkx2.2, Nkx6.2, and Sip1) that associated with oligodendrocyte differentiation, and of proteins (Mog, Mag, Mbp, Mobp, Mal, Cnp, Opalin/Tmem10, Sirt2, and Cldn11) in myelination and ensheathment by the next-generation sequencing analysis on immunoprecipitated spinal cord DNA [40]. Therefore, at least some of the decreased myelin proteins resulted from the downregulation of Olig1 and Olig2 in NPC mice.

However, not all identified myelin proteins are downregulated in this study. We found that only 7 out of the 18 myelin proteins from NPC mice exhibited more than 2-fold reduction when compared to WT mice and the rest had very similar LFQ intensities to WT mice (Fig. 4a). Compared to the proteomic data of different cell types by Sharma et al. [23], the 7 downregulated myelin proteins are exactly the oligodendrocyte-specific proteins that highly expressed in oligodendrocytes but much lower in other cell types (Fig. 4b). Most of the unreduced myelin proteins are also massively expressed in other cell types and plausibly not influenced in NPC mice, therefore, the total amount of these proteins does not present significant differences in the corpus callosum of NPC mice (Fig. 4b).

### Altered sphingolipid-related proteins

Because myelin-enriched lipid is highly required for myelin formation, therefore, its reduction inevitably disturbs myelination, although its shortage is not as destructive as accumulation, as summarized by the previous study from different diseases and animal models [13]. In this study, three sphingolipid-related proteins are downregulated in NPC mice: Cers2, a ceramide synthase [41]; Ugt8, a key enzyme in synthesizing the most abundant myelin lipid - galactosylceramide (GalCer); and Gltp, a transfer protein for various glycosphingolipids between membranes. From the cell type proteomic data [23], Ugt8 and Gltp are exceedingly expressed in oligodendrocytes, while Cers2 is also highly expressed in microglia besides oligodendrocytes (Fig. 4c and d). The defect of Cers2 significantly reduces the compacted myelin and Mbp in the brain of 11-week old mice [42]. Hypomyelinated white matter tracts with unstable myelin sheaths have been reported in the Ugt8 deficient mice [43], however, the transcription and expression of myelin proteins are unaltered [44]. The expression of Ugt8 is positively controlled by Nkx2.2 but negatively modulated by Olig2 [45]. Therefore, besides Olig1 and Olig2, other myelin regulatory signal pathways, e.g., Nkx2.2 pathway, are possibly disrupted in NPC oligodendrocytes.

### The involvement of Gltp during myelination

Gltp is the only downregulated protein that has not been reported whether it is related to myelination or any disease [46]. A decrease of glucosylceramide (GlcCer) level inhibits Gltp expression in glucosylceramide synthase (Ugcg) knockdown cells, while a drug-induced accumulation of GlcCer in a fused endoplasmic reticulum-Golgi complex increases Gltp expression at both mRNA and protein levels [47]. Monohexosylceramides, including GlcCer and GalCer, are reduced by 54.1% in the brain of NPC mice which may result from reduced myelin in the brain, despite massive accumulation in the liver and spleen [48]. Furthermore, the expression of Gltp can only be upregulated by ceramide, but not by other sphingolipids (e.g., GlcCer, GM1, and sphingosine) [49]. We also observed increased Gltp expression in the corpus callosum in WT mice during development (from P8 to adult mice, unpublished data), therefore, its upregulation is conceivably induced by the elevated sphingolipid levels during myelination. The reduction of Gltp in NPC mice may result from the low sphingolipid-contained pre-myelinated oligodendrocytes, which is possibly due to inhibited Ugt8 and Cers2 expression. Moreover, the transcription factor Sp1 (Sp1) regulates the expression of both Mbp and Gltp [49, 50]. Thus, it suggested a potential role of Gltp to participate lipids transport to construct the specially formulated myelin sheaths during myelination, in which its expression was upregulated accompanying with the production of lipid synthase and myelin structural proteins by myelination-related transcription factors. Additionally, the manipulation of the Gltp expression changes cellular lipidome, especially when Gltp is upregulated, both the globotriaosylceramide (Gb3) and GlcCer levels are increased [51] and its overexpression modifies cell shape by interaction with delta-catenin [52]. Therefore, Gltp possibly acts as a sensor to monitor cellular lipid levels and modulates the production of lipid and myelin structural proteins in myelinating oligodendrocytes.

### Upregulated lysosomal and inflammatory proteins

Four proteins are significantly upregulated in the corpus callosum of NPC mice, while neither of them is highly expressed in oligodendrocytes (Fig. 4d). Ctsd and Ctsf are the lysosomal proteases [53, 54] and Hexb forms lysosomal hexosaminidase with Hexa to hydrolyze GM2 [55]. Since lipids, including GM2, are accumulated in the LE/LY and the activity of lysosomal proteases is inhibited in NPC disease, therefore, their upregulation indicates a compensatory effect of the defected LE/LY functions. Additionally, Ctsf is suggested to mediate MHC class II maturation and peptide loading in macrophages [56], and identified in the macrophage-rich areas of the human atherosclerotic lesions and can be secreted by cultured macrophages [57]. Because Ctsf is highest expressed in microglia (resident macrophages in the CNS) (Fig. 4d), its upregulation reflects hyperactive microglia in the disease as reported in NPC mice [58, 59]. The elevated Ctsd was observed in the serum and brain of NPC mice [60, 61]. Furthermore, proteomic analysis by Sleat et al. reveals upregulation of Ctsf, Hexb and other lysosomal proteins in the brain of NPC mice [62]. C1qc and the other 2 components (C1qa and C1qb) form the complement component 1q protein complex (C1q). Besides its function as the initiating protein in the classical complement pathway, C1q also mediates the synapse elimination in the CNS [63]. Furthermore, C1q protein level increases dramatically in the normal aging mouse and human brain, suggesting a linkage to aging-related cognitive decline [64] and the upregulation is also reported in Alzheimer disease [65]. Although increased C1q proteins have been reported in NPC mice [66], abolished C1qa in NPC mice exhibited no improvement, therefore the increased C1qc represents the result of neuroinflammation but not the causes of neurodegeneration in NPC disease [67].

In summary, by the MS-based differential quantitative proteomics, we revealed that the dysfunction of Npc1 is not only associated with a reduced expression of various myelin structural and indispensable proteins (Bcas1, Enpp6, Mbp, and Ugt8) but also the proteins (Cers2, Ugt8, and Gltp) related to sphingolipid metabolism in NPC mice. Furthermore, the involvement of Gltp during myelination is proposed. Besides myelin sheaths, the proteome of the corpus callosum contains proteins from other cell types that contribute to myelination, therefore, the use of the corpus callosum or similar structures is suggested to elucidate protein dynamics from different cell types during myelination.

## Additional file

Additional file 1: The validated proteins/protein groups that identified in all samples and their UniProt accessions, protein and gene names, Log2 LFQ intensities and the number of peptides from each sample. (XLSX 539 kb)

## Abbreviations

Aldh1l1: Cytosolic 10-formyltetrahydrofolate dehydrogenase
Aqp4: Aquaporin-4
Bcas1: Breast carcinoma-amplified sequence 1
C1qc: Complement C1q subcomponent subunit C
Cers2: Ceramide synthase 2
Clc4: Chloride intracellular channel protein 4
Cldn11: Claudin-11
Cnp: 2′,3′-cyclic-nucleotide 3′-phosphodiesterase
CNS: Central nerves system
Ctsd: Cathepsin D
Ctsf: Cathepsin F
DTT: Dithiothreitol
Enpp6: Ectonucleotide pyrophosphatase
FA: Formic acid
FASP: Filter-aided sample preparation
FDR: False discovery rate
GalCer: Galactosylceramide
Gb3: Globotriaosylceramide
Gfap: Glial fibrillary acidic protein
GlcCer: Glucosylceramide
Gltp: Glycolipid transfer protein
Go BP: Gene ontology biological processes
Go CC: Gene ontology cellular compartment term enrichment
Hexb: Beta-hexosaminidase subunit beta
IAA: Iodoacetamide
LE/LY: Late endosome and lysosome
LFQ: Label-free quantification
Mag: Myelin-associated glycoprotein
Mbp: Myelin basic protein
Mog: Myelin-oligodendrocyte glycoprotein
MS: Mass spectrometry
Myrf: Myelin gene regulatory factor
Nkx2.2: Homeobox protein Nkx-2.2
NPC: Niemann-Pick Type C disease
Npc1: NPC intracellular cholesterol transporter 1
Npc2: NPC intracellular cholesterol transporter 2
Olig1: Oligodendrocyte transcription factor 1
Olig2: Oligodendrocyte transcription factor 2
P: Postnatal day
Pbdc1: Protein of PBDC1
Plp: Proteolipid protein
Plxnb3: Plexin-B3
Rhog: Rho-related GTP-binding protein RhoG
Sirt2: NAD-dependent protein deacetylase sirtuin-2
Sox10: SRY-related HMG-box 10
Sp1: Transcription factor Sp1
SQS: Squalene synthase
Ugcg: Glucosylceramide synthase
Ugt8: UDP glycosyltransferase 8
UPLC: Ultra Performance liquid chromatography
WT: Wildtype
